# Why do participants drop-out: findings from a prospective pediatric cohort for fever surveillance established at Vellore, southern India

**DOI:** 10.1186/s12874-019-0881-y

**Published:** 2019-12-30

**Authors:** Kulandaipalayam Natarajan Sindhu, Manikandan Srinivasan, Sathyapriya Subramaniam, Anita Shirley David, Venkata Raghava Mohan, Jacob John, Gagandeep Kang

**Affiliations:** 1 Wellcome Trust Research Laboratory, Division of Gastrointestinal Sciences, Christian Medical College, Vellore, 632004 India; 2 Department of Community Health, Christian Medical College, Vellore, 632002 India

**Keywords:** Cohort, Longitudinal, Drop-out, Pediatrics

## Abstract

**Background:**

Cohort studies are pivotal in understanding the natural history, and to thereby determine the incidence of a disease. The conduct of large-scale community-based cohort studies is challenging with reference to money, manpower and time. Further, attrition inherent to cohort studies can affect the power, and thereby the study’s validity. Our objective was to estimate the percentage of participant withdrawal and to subsequently understand reasons for the same in the Vellore Typhoid Surveillance (VTS) cohort.

**Methods:**

VTS study, a prospective community-based pediatric cohort, was established in a semi-urban settlement of Vellore to estimate the incidence rate of typhoid fever. An active weekly surveillance identified children with fever, and blood cultures were performed for fevers of ≥3 days. Reasons for participant drop-out in the cohort were documented. Nine focus group discussions (FGD), each with 5 to 7 parents/primary caregivers of former as well current participants were conducted separately, to understand reasons for consent withdrawal as well as the good aspects of the study that the current participants perceived. A descriptive, as well as an interpretative account of the themes that emerged from the FGDs were done.

**Results:**

Of the 5639 children in the VTS cohort, 404 (7.2%) withdrew consent during the 12-month surveillance. Of these, 50% dropped out due to migration from study area; 18.1% as their parents were unhappy with the blood draws for blood culture; and 14.4% did not clearly put forth the reason for consent withdrawal. Being from an orthodox background, high socio-economic status and joint family were associated with a decision to drop-out. Frequent and voluminous blood draws, male field research assistants (FRA) making weekly home-visits, the perception that inquiring about fever made their child fall sick, and that the study clinic did not initiate antibiotics immediately, were the important themes that emerged from the FGDs conducted among drop-outs.

**Conclusion:**

Our study showed that specific beliefs and behaviours within the community influenced the drop-out rate of the VTS cohort. Background characteristics and perceptions that exist, along with attrition data from previous cohort studies in the specific community are important to be considered while implementing large-scale cohort studies.

## Background

A cohort study design is an essential and a pivotal epidemiological tool to study the natural history of a disease and thereby, deduce its incidence. Cohort study designs have the added advantage of studying multiple risk-factors and is not possible with other study designs [1]. However, the implementation of, and running a cohort study can be enormously resource constraining in terms of money, manpower and time, and hence requires implementation with utmost meticulous planning. The results of cohort studies are valuable in that the results can be generalized to the wider population; however, it is imperative that the study sample be representative of the population of interest. Hence, the cohort must not be subject to selective attrition that can bring in glaring differences in the characteristics between the subjects who dropped out and those who completed follow-up. Attrition can significantly affect the power and validity of the study, and further the statistical analysis [2]. Hence, with regards to attrition, understanding the characteristics of the drop-out population from within a cohort is important not for just for the statistical analyses, but to understand the weakness of the study and challenges faced in the follow-up of the cohort, thereby being valuable as essential learning points that would go on to be crucial while implementing future cohort studies in the community.

A wide array of factors influence participant withdrawal in large-scale community-based longitudinal studies. Young mothers, poor literacy, being from the low socioeconomic strata or minority race/ethnicity, poor participation from the father and the participant’s poor health status are some of the factors known to influence low response and drop-out rates in longitudinal studies, and these factors seem to be relevant in the context of both high-income as well as the low-and-middle countries, though studies documenting attrition rates and reasons for the same in LMIC are very few in numbers [3–8]. Further, mothers who perceive their children to be at high-risk for the disease being investigated in the study and who fall sick more often seem to show better retention rates in cohort studies [4]. Added to the impact of socio-demographic parameters on the retention rates in cohort studies, is the dimension of psychological and behavioural aspects of participants that is off lately being increasingly recognized [4].

The process of informed consent is administered in accordance with three important elements – providing adequate information on the research study for decision making, comprehension, and voluntariness [9]. Though the contents of the informed consent form (ICF) are under rigorous scrutiny by the ethical committee, the magnitude and quality of understanding at the participant level, that is the element of “comprehension” is difficult to assess and measure, and further challenging to validate [10, 11]. Obtaining an informed consent meticulously in large cohort studies, where time constraints for enrolment stand in way with reference to pre-protocolled timelines, can make the process highly challenging. Participants’ internalization of the research study, and understanding its future implications for them as individuals, as well as their community and country, on the whole, is difficult to ascertain. This is a bigger challenge especially in the Indian setting given that the internalization and understanding is influenced by numerous factors such as language congruence between the researcher and the subject, socio-economic status, literacy, religious background and importantly, the prevailing social inhibition to question the healthcare professional about the research study during the consenting process [10]. Literature shows that participants in developing countries hesitate to decline research participation when approached, with the perception or anxiety that doing so could potentially hamper their routine clinical care at the concerned healthcare facility [12]. Further, a meta-analysis has shown that reasons that motivate parents to consent for participation of their children in research included receiving better access to healthcare as a result of study participation, financial reimbursements and the benefit of having the medical team contact or visit them without delay [13]. Hence, there exist multiple invisible factors that blanket the informed consenting process in the background and can hugely influence the participant’s understanding and attitude towards research. In addition, importantly in large-scale cohort studies, it is pivotal to understand the population dynamics, behaviours and perceptions within the frame of their cultural norms. This is specifically important to be considered in heterogeneous communities with different religions, castes or ethnicities staying together, to enable follow-up with minimal attrition. Hence, this iterative process of incorporating cultural sensitivity and awareness of population behaviour is necessary within the cohort implementation teams to enhance the robustness of large-scale, community-based longitudinal studies [14].

Our observations and qualitative discussions are based on the Vellore Typhoid Surveillance (VTS) study, a community based pediatric cohort set up in a semi-urban settlement of Vellore. Our study aimed to estimate the percentage of subjects who dropped out of the VTS study and to compare the baseline characteristics of subjects who dropped out with those who continued participation in the study. Further, focus group discussions (FGD) were employed to understand reasons for consent withdrawal among the parents/primary caregivers of children who dropped out.

## Methods

### Study setting, design, and population

VTS study was established in October 2016 at Vellore. Vellore city (12.92°N 79.13°E) in Vellore district, is one of the 32 districts situated in Tamil Nadu, the southernmost state of India [15]. VTS study was a prospective community-based study in which a pediatric cohort was established to estimate the incidence rate of fever, specifically typhoid fever. This closed cohort comprised of children aged between one and 15 years residing in the four contiguous semi-urban settlements of Chinnallapuram (CAP), Kaspa (KASPA), Ramnaickanpalayam (RNP) and Vasanthapuram (VSPM). The four areas comprise of approximately 10, 000 households with a population of 42, 000. About 15% of the population comprises of children aged less than 15 years. The population is dependent on income through daily wage-based jobs, with ‘beedi-work’ (an indigenous cigarette made with unprocessed tobacco) being the predominant occupation [16]. Urban health centers in the area that are state-run provide healthcare free-of-cost to the residents within the area, with a government teaching tertiary-care hospital located at a radius of ∼5 km. Christian Medical College (CMC), Vellore, a not-for-profit organization with its two outreach hospitals - the Community Health and Development (CHAD) hospital and the Low Cost-effective Care Unit (LCECU), are situated within a few kilometers from the study area [17]. The population of the area also access healthcare provided by private general practitioners and private nursing homes located in-and-around the area. The Wellcome Trust Research Laboratory under the Division of Gastrointestinal Sciences, CMC has been closely working with this population over the last 18 years and has been providing pediatric outreach care services through a clinic, the Chinnallapuram (CAP) community clinic that was established in 2002.

**Fig. 1:**
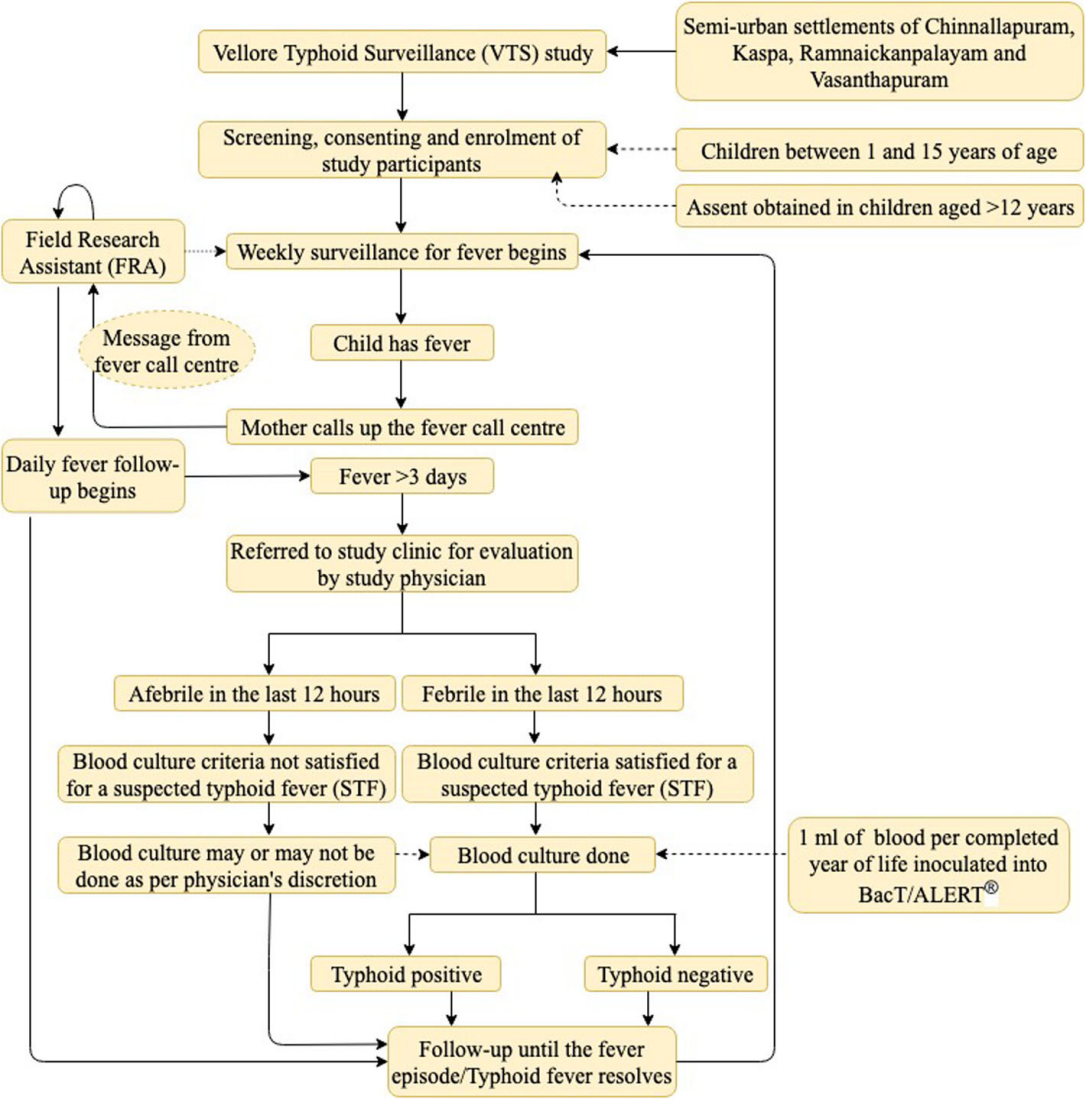
Schematic representation of the flow process of Vellore Typhoid Surveillance (VTS) study

A Vellore Health Demographic and Surveillance (HDSS) system has been established in the area by the Wellcome Trust Research Laboratory. An Eligible Child Register (ECR) was generated from the HDSS, to identify the eligible households with children aged between one and 15 years. Following the dissemination of information about the study to the community and the community leaders, the households with eligible children were invited to participate in the study. The inclusion criteria to participate in the surveillance included the child being available in the study area for at least one year and willing to permit weekly phone call/home-visit by a Field Research Assistant (FRA). Those with profound mental or physical disabilities or planning to potentially migrate from the study area were excluded from the study. Following the screening that was done by the FRAs, a written informed consent (and assent from children aged more than 12 years) was obtained from the parent/primary caregiver of the child after having explained the study details and procedures. The flow process of the VTS study is shown in Fig. 1.

Following enrolment, weekly surveillance was initiated by the FRA where the parent/primary caregiver in the household would be asked for a history of fever and other illnesses in the child over the last seven days. A prior pilot study done before the implementation of the VTS revealed that parental report of fever, either through phone call or by a home visit by the FRA, was the most feasible and reliable way to capture fever in their children in this community. Overall, 19 FRAs conducted surveillance for the four areas under the supervision of two Field research supervisors (FRS). Also, parents/primary caregivers were requested to notify the central fever call center, established specially for the VTS study, in case the child was running a fever. Following this, the call center would pass on the message to the concerned FRA, who would then initiate a daily fever follow-up. If the child continued to remain febrile for more than three days or if the child was sick, FRA would issue a referral slip to visit the CAP clinic. A child was deemed to have suspected typhoid fever (STF) if the child had a fever for more than three continuous days. Following evaluation by the physician at the study clinic, and the fulfillment of the STF criteria, a blood culture request was made if the child was febrile in the last 12 h. Blood culture was performed by a trained phlebotomist by inoculating 1 ml of blood per completed year of age of the child into BacT/ALERT® culture media bottle [18]. Parents/primary caregivers were also free to seek care from private practitioners of their choice, with the practitioners being requested by the study team to refer the child to CAP clinic for a blood culture. In such cases, culture reports, both a preliminary and a final report, were issued back to the concerned parent/primary caregiver so that they could be followed-up and treated accordingly by their treating physician. Medications for children who visited CAP community clinic were prescribed accordingly and antibiotics were prescribed as per the Hospital infection control committee (HICC) antibiotic guidelines for paediatrics issued by Christian Medical College, Vellore. Following culture and additional blood tests as requested by the physician such as blood counts, the child was further followed up until the child became afebrile for 48 h following the last fever spike. If the blood culture grew *Salmonella Typhi/Paratyphi,* the child was seen again by the physician and was started on antibiotics as per the protocol for the management of typhoid fever. If the study physician felt the child was sick and needed hospitalization, the child was referred to CMC/CHAD/LCECU depending on the severity of the illness. The expenses incurred towards diagnostics and treatment of all children with fever visiting the CAP clinic, including those diagnosed with typhoid fever, were borne by the study. Also, expenses incurred towards any fever-related hospitalization at CMC/CHAD/LCECU was taken care of by study during this period.

### Data collection

FRAs completed the weekly fever surveillance forms using electronic tablets with an in-house developed android app, and the data captured by the FRAs was synced at the end of every day to a centrally managed cloud-based infrastructure. Weekly surveillance was done by a physical visit once a month, with surveillance for the other three weeks of the month being done by either a phone call or a home-visit. A surveillance initiated for a fever mandated a physical visit every day until 48 h following the resolution of fever. Data captured included the start date of fever, the end date of fever, highest temperature, outpatient visit or hospitalization details, antibiotics used during the course of the episode and other illnesses. In cases where the family migrated from the study area, or there was a loss to follow-up or withdrawal from the study, a censorship form was filled by the FRA for the child/children following a waiting period of 4 weeks and confirmation by the FRS. In cases where the parent/primary caregiver withdrew consent or did not want to continue the child/children in the study, a home visit was first made by the FRS within 2 weeks. A detailed report was made by the FRS following which the report was validated by the senior research officer (SRO) by a home-visit and reason for the consent withdrawal was documented.

### Focus group discussions (FGD)

In 17.9% of former participants where it was difficult to establish a concrete reason for the decision to withdraw from the study, facilitator-based FGDs were conducted in the community among the parents/primary caregivers of children who dropped out, to elicit and understand the probable sensitive background issues that could have been the reason for consent withdrawal. The discussions were conducted in all the four areas with the facilitator moderating the session and a scribe taking notes of the discussion. A female facilitator engaged in moderating the sessions in areas where women were hesitant to interact with a male facilitator. FGDs were also conducted among parents/primary caregivers of children who were current participants in the study to understand the attitude and perceptions regarding the study, strengths of the study and overall, the attitude towards research in the community.

A verbal consent was obtained from both the drop-out as well as the currently participating mothers of the VTS study, for their participation in the FGDs. Further, consent was also taken to audio-record the FGD, and since the majority of the mothers, especially those from orthodox backgrounds, were not happy with their opinions being recorded, no audio-recording was employed for the FGDs. A scribe recorded the conversations between the mothers and the facilitator in the local language, Tamil. Names of the mothers were not captured and instead subject numbers such as subject 1, 2 and so on were used for each FGD. Overall, nine focus group discussions (FGD), each with 5 to 7 parents/primary caregivers of former as well current participants were conducted separately.

### Statistical analysis

Categorical variables that included socio-demographic characteristics, dropout rates and reasons for drop-out were expressed as percentages. Chi-square test for proportions was used to compare socio-demographic variables across the four areas, and also to compare the characteristics between the two groups: children who dropped out and those who continued participation in the cohort. A *p*-value of less than 0.05 was considered as the level of significance.

A transcript-based analysis was performed for the FGDs. Notes taken by the scribe from the FGDs were transcribed from Tamil to English, thereby generating transcripts. The contents of the transcripts were grouped under headings based on similarities in the responses that emerged from the FGDs, which were coded. These codes generated were further clubbed to themes. Quotations within the themes identified were used for the narrative summary. Two raters independently identified the themes that emerged from the FGDs. Conflicts or discrepancies in the themes generated were resolved by a third rater. Group dynamics such as hesitancy to open up during the initial part of the FGD, shy participants and facial grimaces were noted as observed by the facilitator. The questions used in the FGDs for both the former as well as current participants of the VTS study are shown in Table 5.

## Results

### Background characteristic of the study area in which the VTS study was established

The characteristics of the overall population of the study areas namely Chinnallapuram (CAP), Ramnaickanpalayam (RNP), Kaspa (KASPA) and Vasanthapuram (VSPM) are depicted area-wise in Table 1. It can be seen that the four areas are significantly different from one other with reference to the age distribution of the children, type of family, religion, SES, and education including mother’s education. Majority of the families residing in the four areas were of the nuclear family type (71.8%) and predominantly belonged to the middle-class of socio-economic status (56.3%), with a third belonging to the lower socio-economic strata. Hindus and Muslims form 49.3 and 45.6% of the population respectively.

**Table 1:** Characteristics of the population in the four settlements of the study area *[N = 42, 177 (10, 102 households)]*

		Total		CAP		KASPA		RNP		VSPM		*p*-value
		*n*	*%*	*n*	*%*	*n*	*%*	*n*	*%*	*n*	*%*	
Total population^#^		42,177		11,899	28.2	12,788	30.3	10,339	24.5	7151	17	–
Population aged < 15 years^#^		10,149	24.1	2999	29.5	2863	28.2	2622	25.8	1665	16.4	–
Age distribution of children	< 5 years	3051	30.1	965	32.2	840	29.3	759	28.9	487	29.2	0.041
	5–10 years	3574	35.2	1053	35.1	1016	35.5	938	35.8	567	34.1	
	10–15 years	3524	34.7	981	32.7	1007	35.2	925	35.3	611	36.7	
Number of families^#^		10,102		2889	28.6	3117	30.9	2338	23.1	1758	17.4	–
Type of family	Nuclear	7254	71.8	2184	75.6	2181	70	1618	69.2	1271	72.3	< 0.0001
	Joint/extended	2848	28.2	705	24.4	936	30	720	30.8	487	27.7	
Religion	Hindu	4981	49.3	1554	53.8	1782	57.2	234	10	1411	80.3	< 0.0001
	Muslim	4612	45.6	1281	44.3	1037	33.3	2071	88.6	223	12.7	
	Christian	503	5	52	1.8	297	9.5	31	1.3	123	7	
	Others	6	0.1	2	0.1	1	0	2	0.1	1	0	
Socio-economic status	Low	2975	29.4	930	32.2	784	25.2	678	29	583	33.2	< 0.0001
	Middle	5689	56.3	1558	54	1865	59.8	1272	54.4	994	56.5	
	High	1438	14.3	401	13.8	468	15	388	16.6	181	10.3	
Education (highest in household)	No education	575	5.7	201	7	141	4.5	122	5.2	111	6.3	< 0.0001
	Class 1 to 5	555	5.5	198	6.9	127	4.1	156	6.7	74	4.2	
	Class 6 to 8	1429	14.1	488	16.8	324	10.4	400	17.1	217	12.3	
	High school	2626	26	809	28	778	25	573	24.5	466	26.5	
	Higher secondary	1886	18.7	520	18	599	19.2	446	19.1	321	18.3	
	Degree	3031	30	673	23.3	1148	36.8	641	27.4	569	32.4	
Mother’s education	No education	4783	47.3	1405	48.6	1388	44.5	1113	47.6	877	49.9	< 0.0001
	Class 1 to 5	1260	12.5	394	13.6	314	10.1	406	17.4	146	8.3	
	Class 6 to 8	1590	15.7	460	16	510	16.4	377	16.1	243	13.8	
	High school	1427	14.1	387	13.4	519	16.6	239	10.2	282	16	
	Higher secondary	581	5.8	144	5	214	6.9	123	5.3	100	5.7	
	Degree	461	4.6	99	3.4	172	5.5	80	3.4	110	6.3	

*CAP*: Chinnallapuram; KASPA: Kaspa; RNP: Ramnaickanpalayam, VSPM: Vasanthapuram

^**#**^ Row percentage

^*^ Socio-economic status was classified as low, middle and high using the modified Kuppusamy scale that included education, occupation and selected assets [19]

Of the four areas, RNP had the highest proportion of Muslim population (88.6%), with the highest proportion of joint/extended families (30%) when compared to the other three areas. Nearly, 17% of this population belonged to the high SES strata, the highest among the four areas. Next to RNP, CAP had a significantly higher proportion of Muslims (44.3%) with the majority of the families belonging to the nuclear family type (75.6%). About 32% of its population belonged to low SES. Kaspa, on the other hand, had only one-third of its population being constituted by Muslims, significantly less when compared to CAP and RNP, with one-third of its families living as joint/extended families. Kaspa had the highest proportion of its population (60%) belonging to middle SES. VSPM had a predominantly Hindu population (80%) when compared with the other three areas. Similar to CAP, the area had predominantly families belonging to the nuclear family type (72%), with a considerable percentage belonging to the low SES background (33%). To summarize, RNP had the highest proportion of Muslims, families which were joint/extended and belonging to high SES. On the other hand, CAP also had a considerable proportion of the Muslim, but with its population predominantly from the low SES background. Also, CAP had a higher proportion of nuclear families.

Overall 5639 children, aged between 1 and 15 years were enrolled to participate in the VTS study. Of the 5639 children who participated in the surveillance, 404 (7.2%) dropped out of the study during the 12-month follow-up period. Baseline characteristics of children who dropped from the study were compared with those who continued participation in the study (Table 2). A higher proportion of drop-out children were from the Muslim background (52.5% vs. 47.5%, *p = 0.211*), however, this was not statistically significant. Also, the drop-out group had a significantly lesser proportion of people belonging to low SES when compared to the group who retained in the study (61.6% vs. 67.2%, *p* = 0.001). Further, drop-outs from joint/extended families were more likely to drop-out than the participants who continued (51.5% vs. 38.7%, *p* < 0.0001). Children who dropped out of the study had a lesser proportion of mothers with low educational status (class 5 and below) when compared to those who continued participation (49.3% vs. 52.3%, *p* = 0.094), however, this was not statistically significant. Drop-out rates calculated were adjusted for area-wise contribution to the cohort enrolled (Table 3). Following this adjustment, it was seen that RNP had the highest drop-out rate of 2.2% when compared to VSPM which had the lowest (1.3%).

**Table 2:** Comparison of baseline characteristics of participants who dropped out with participants who continued participation in the study *(N = 5639)*

		Participants who dropped out *(n = 404)*		Participants who continued in the study *(n = 5235)*		*p*-value^†^
		*n*	*%*	*n*	*%*	
Religion	Hindu	166	41.1	2518	48.1	0.016
	Muslim	212	52.5	2485	47.5	0.211
	Christian	26	6.4	232	4.4	ref
Socio-economic status*	Low	249	61.6	3520	67.2	< 0.0001
	Middle	130	32.2	1556	29.7	0.006
	High	25	6.2	159	3	ref
Mother’s education	Below class 5	199	49.3	2740	52.3	0.094
	Class 6 to 10	154	38.1	1961	37.5	0.246
	Class 11 & above	51	12.6	534	10.2	ref
Type of family	Nuclear	196	48.5	3208	61.3	ref
	Joint/extended family	208	51.5	2027	38.7	< 0.0001
Age at drop-out	1 to 5 years	106	26.2	1579	30.2	ref
	5 to 10 years	168	41.6	2152	41.1	0.239
	10 to 15 years	130	32.2	1504	28.7	0.062
Area-wise drop-out rate^#^	CAP	85	5.7	1414	94.3	ref
	KASPA	121	6.7	1685	93.3	0.223
	RNP	123	8.4	1337	91.6	0.003
	VSPM	75	8.6	799	91.4	0.006

^†^Chi-square test was used as test of significance between the two proportions

^#^ Row percentage

^*^ Socio-economic status was classified as low, middle and high using the modified Kuppusamy scale that included education, occupation and selected assets [19]

**Table 3:** Area wise drop-out rates adjusted to area-wise contribution to the VTS study cohort enrolled *(N = 5639)*

Area	Number of children enrolled	Area-wise contribution to the cohort enrolment	Drop-out rate	Drop-out rate adjusted to area-wise contribution to the cohort enrolled
CAP	1499	26.6	5.7	1.5
KASPA	1806	32	6.7	2.1
RNP	1460	25.9	8.4	2.2
VSPM	874	15.5	8.6	1.3

Of the 404 (7.2%) children who dropped out, a major proportion of children (50%) dropped out of the study as a result of migration from the study area (Table 4). Further, 18.1% of the children dropped out of the study, with their parents stating the reason that they were unhappy with the blood draws that was performed for a blood culture. Another 14.3% of the children dropped out with parents unwilling to continue their participation in the study, and in a majority of these cases, it was difficult to elicit the reason for consent withdrawal by the FRA/FRS.

**Table 4:** Reasons for dropouts as recorded at the time of censorship in the Vellore Typhoid Surveillance (VTS) study *(n = 404)*

Reason	*n*	*%*
Family migrated from the study area	202	50
Unwilling to continue in the study because of blood draw	73	18.1
Parent’s/primary care-givers unwilling to continue in the study for reasons not specified	58	14.3
Not available for follow-up for next one year (soon to migrate)	57	14.1
Child admitted at boarding school away from the study area	14	3.5

### Qualitative summary of the focus group discussions (FGD)

Five FGDs were conducted with the parents/primary caregivers of children who dropped out of the study, and four FGDs with those who continued participation in the study (Table 5). Each FGD involved five to seven parents/primary caregivers, and all the participants in the FGD were women. Two FGDs among the former participants comprised of only three women, with no response from the others who were invited to participate in the discussion for the day. A total of 31 mothers of former participants were invited, of whom 6 declined to participate. Among 21 mothers of the current study participants who were invited, all 21 took part in the discussion.

**Table 5:** Qualitative summary of the focus group discussions (FGD) conducted among parents/primary caregivers of the children who dropped out and from the study

FGD question	Response of the participants
*What were the reasons that influenced your decision to withdraw your child from the study?*	*“FRA visiting home/calling every week asking about fever is too much; FRA in previous studies contacted us only once-a-month.”*
	*“My husband dislikes male FRA visiting our house.”*
	*“As soon as the FRA inquires about any fever, my child immediately falls sick.”*
	*“Too much of blood is being drawn from my child for a blood test.”*
	*“Collecting blood from my child for testing makes my child very weak.”*
	*“My child became weak after the last blood test.”*
	*“Study clinic initiates antibiotics for my child only after three days of fever whilst private practitioners initiate antibiotics and gives injections*^*†*^ *early, and hence recovery is faster with care from a private practitioner.”*
	*“At the time of consenting, I was not aware that a blood test would be requested if my child has a fever for more than 3 days.”*
	*“My daughter has attained puberty.”*
	*“My child’s school is strict and does not permit leave for my child to visit the study clinic for a blood test.”*
	*“There is no specific reason, but my husband does not want our child to participate in the study further.”*
*Did you read and understand the information sheet before signing the consent form?*	*“I am not sure if I read the information sheet before consenting.”*
	*“I did not read the information sheet, but my husband did.”*
	*“I read and understood the information sheet and consented, however, I later changed my mind as I did not want my child to be subjected to the voluminous and frequent blood tests.”*
	*“Yes, I read the information sheet and signed it, as I thought it is good for my child to be in the study no matter what.”*
*Did you discuss with husband/family members before enrolling your child in the study?*	*“No, I did not discuss with my husband or family members before joining the study. I decided to enrol my child in the study on my own and hence consented.”*
*According to you, what were the good aspects of the study?*	*“The FRA advised us to boil water and drink at all times especially during the rainy season, and that prevented my family members from falling sick too often.”*
	*“FRAs taught us the importance of wearing foot-wear, washing vegetables before use and handwashing.”*
*Do you think research is helpful to your community?*	Yes, previous studies in the area helped bring in a new vaccine* by the government to protect children from diarrhoea.
*Will you participate in future research studies if conducted in your area?*	*“Yes, we will participate provided there are no blood tests in children.”*
	*“We will decide when a study comes up in the future.”*
	*“No, I will not participate as my husband is against research studies on my child.”*

† unknown injections *Rotavirus vaccine

Following the initiation of the FGDs among mothers/primary caregivers of former participants, it was observed that most of the mothers were quiet, looked anxious and did not interact or respond to the questions. On reassuring the mothers that this exercise was to understand the study and community better and that this discussion will in no way affect their healthcare-seeking at CAP clinic, a few mothers started conversing followed by the others. Three important themes emerged from the FGDs involving the mothers of former participants, from the reasons put forth by them as to why they chose to drop-out from the study. Firstly, mothers/caregivers were unhappy with the FRA making a home visit for fever surveillance almost every week, especially with the Muslim mothers who come from conservative backgrounds in the community. Some mothers were unhappy with surveillance even through phone calls by male FRAs. It was learned that the spouses of these women were not very happy when male FRAs visited their homes for weekly surveillance. This occasionally led to conflicts with the husband and with household members, especially the elders in joint families. Also, it emerged that a perception eventually built up over time that every time the FRA inquired about fever, whether as a home-visit or a phone call, the child fell sick. The second theme that emerged, and that was profoundly emphasized by the mothers was about the venipuncture that was performed for blood culture in cases of STF. Mothers felt that blood being drawn to be inoculated into the BacT/ALERT bottle was quite voluminous for just a single test, and believed that it subsequently made the child physically weak. Also, with every episode of fever, whenever the child fulfilled the STF criteria, given that a blood culture was done for a recent episode, parents/caregivers were unhappy on the same being requested again, saying the blood draws for blood culture was apparently too *“frequent” and “voluminous’*. Thirdly, it was felt that the study clinic never initiated antibiotics for fever within the first 2 or 3 days, whereas when they sought care from a private practitioner, they easily had antibiotics prescribed and even got injections on day one of fever, which apparently made recovery faster for their child.

It was surprising to hear that certain mothers who were initially keen on participating in the study said they were not aware that blood tests would be requested every time their child had a fever. Others put forth that they wanted to be a part of the study but the school of the child did not permit time in between for the child to visit the clinic during working hours for a blood culture and hence felt there was anyway no point in taking part in the study as they would not be contributing much. One mother put forth was that her daughter had attained puberty and hence would not permit the child coming out to visit the clinic for blood tests, as their family forbid girls going out of the house frequently after having attained menarche.

With reference to having read and understood the information sheet before consenting, a majority of the mothers responded that, as their husbands had read the information sheet, they felt that there was no necessity for them to read the same again. Some mothers put forth that they did read the information sheet in detail and consent for participation, but withdrew the consent later quoting the too *“frequent”* and “*voluminous”* blood draws from their child as the reason that made them unhappy. A few responded that they read the information sheet and decided to enrol the child in the study, thinking that participating in the study was anyway good for their child, irrespective of what the study was about. Some mothers said they did not discuss the study with their husbands, and enrolled the child in accord with their own decision.

Some notable positive aspects of the study as perceived by these mothers were discussed. Mothers felt that the health-related information they received while interacting with the FRAs helped them induct certain healthy behaviours in their households. Hand washing and boiling water before consumption were the notable and common points put forth. When questioned whether previous research studies in their area had any impact on them or the community, mothers said that a large-scale vaccine study in their community a few years ago had brought about a vaccine (Rotavirus vaccine) being included in the national immunization program, and they welcomed and appreciated this. A mixed response was noted when mothers/primary caregivers were asked if they would participate in future research studies in the area, with some saying they would participate if there were no blood draws on children and others saying they would think about participation maybe when a study comes up in the area. Very few mothers said they would not participate in future studies as their husbands were against research in the area. One mother opined that *“research is meant only for the poor”* and hence she did not wish to participate.

FGDs among parents/primary caregivers of children who are currently participating in the study appreciated the services rendered by the study clinic and said the physicians prescribed *“milder”* medicines when the child was sick. One mother said, *“It is dangerous to give antibiotics too often to children, and hence was happy that the study clinic does not prescribe antibiotics easily”.* The mothers were happy with blood tests being done but wished blood tests for other fevers were also performed. Also, they liked interacting with the FRAs who often gave them good advice related to hand washing and hygiene. On being asked what this research study meant to them, all the mothers unanimously said they did not know about the benefits of this research study to the community, but that it certainly was beneficial to them individually as their child was taken care of by the study team. All the participants in this FGD said that they had read the information sheet and discussed this study with their husbands before consenting.

## Discussion

The Vellore Typhoid Surveillance (VTS) study documented a drop-out rate of 7.2% at the end of 12-month follow-up. This is comparatively low when compared to other pediatric cohorts from studies of western countries which have reported drop-out rates between 14 to 35% [4–6]. However, further comparison of these drop-out rates to our study is limited by the varying follow-up periods in these cohorts. Literature documenting attrition data from LMIC countries is very limited and hence, it was challenging to make comparisons with cohorts from settings similar to ours. Nevertheless, we documented the drop-out rate and exercised efforts to understand reasons for drop-outs in the VTS cohort, a large pediatric cohort in southern India, making it one of the very few studies documenting the same.

Our observations from the cohort of Vellore Typhoid Surveillance study have several aspects to note, most important being that this was not just a heterogeneous population in terms of socio-economic status, religion, income and educational status, but also with reference to attitude and perceptions of the population towards the study and research given the background the participants are from. It was observed that orthodox communities such as the Muslim community showed a higher drop-out rate in the cohort. Also, children from joint or extended families had a higher probability of dropping out. This is a community where traditional joint families still exist in significant proportions. The routine consenting process involves only the parents of the child, with family members in joint families not being involved, in the least sensitizing them about what the study is about and its impact. Developed countries have just initiated steps in the implementation of “engagement science’ where family members are also being engaged in research studies [31]. This, though promising, is yet to be conceived as a good tool in the implementation of large-community based cohorts in developing countries given the limited resources with reference to time and money in research. Also, it was seen that participants dropped out significantly from the higher strata of SES, and it was difficult to understand the reason behind this. One possible explanation is through a cue picked up from the FGD that some considered research being meant for the poorer sect of the community and not for the well to do, though this was not put forth openly by all mothers in the discussions.

FGDs played a pivotal role in understanding the sensitive reasons behind dropping out of the VTS study especially from within certain pockets of the community. Male FRA visiting orthodox families for fever surveillance brought about dissent within some families, however, this being the reason for consent withdrawal was not put forth directly when inquired by the FRS as the parent/caregiver did not want to offend the FRA/FRS. The biggest challenge that was visible from the FGDs with the former participants was that parents/primary caregivers could not perceive the importance of collecting an adequate blood sample for a blood culture. WHO emphasizes the volume of blood being critical in isolating *Salmonella Typhi/Paratyphi* and recommends 10–15 ml of blood from children aged more than five years; and 2–4 ml from preschool children and those even younger [20]. Parents felt too much of blood (2 ml in preschool children, and 5 ml in school going children) was collected *“unnecessarily”* for just a single test, whereas private practitioners collected very little blood and did tests delivering the results instantly, while blood culture reports required a waiting period of at least 3 days. It was learned that Widal and other rapid tests, that lack specificity in typhoid endemic settings like India, were very commonly performed by the private practitioners in this area. Blood culture as the gold standard for typhoid fever was not considered in practice in this area [21, 22]. This was because setting up a blood culture facility needed huge technical and manpower investments by the practitioners. The existing disparities in diagnostic methods for fever among the general practitioners in this area often saw parents/primary caregivers disagreeing to blood cultures done at the study clinic, as this was something they were not aware of. Further, as documented in a study in Nigeria, blood draws at the study clinic for blood culture triggered spouse disapproval of the same with occasional conflicts, leading to consent withdrawal [23]. Previous studies done in the same area were successfully able to sample infants and children, however, these samples were at specific time-points that the parents were aware of right from the beginning of the study. However, VTS being the first-time largest cohort in this community, the protocol that called in for blood sampling as and when the child had fever was new to this community, and parents were not used to this kind of a study.

Another reason noted for drop-out through the FGDs was that the study clinic did not initiate antibiotics for fevers immediately. Studies in the past from Vellore have shown antibiotic prescription rates to be high for patients presenting with fevers, and this was highest in the pediatric population [24, 25]. This is an even bigger challenge with the fact that irrational antibiotic usage in the community is high and perhaps future antimicrobial stewardship and IEC (information, education, communication) programs could herald positive changes in the community. However, the road to this is long and addressing this to improve cohort retention is not easy.

Another important aspect that emerged was that mothers felt they didn’t need to read the information sheet in detail, as their husbands had read the same before consenting. This is again a challenge in settings like these where the mother is the primary caregiver. Further, how far can the research team go in delivering a *“truly understood”* consent is challenging. This perhaps demands the team spending a significant amount of dedicated time with the parent or primary caregiver, and make use of simple yet useful methods such as making the participant repeat-back information or answer questions related to the study [26, 27]. In huge community-based cohorts, given the timelines to meet and constrained resources, this can be quite arduous. Also, this can be difficult given that in a heterogeneous community such as this, the reciprocation and understanding, with the internalization of the impact these studies have on their community, can vary hugely which means spending different lengths of time with different subjects. To tailor this for the person delivering the consent accordingly may be way beyond the capacity in large community-based studies. Also, our study was non-incentive based except that the team took care of fever-related treatment and hospitalizations during the study period. Research using incentives based cohort retention have proved successful [28]. However, from the ethical angle, is this valid and justified is debatable, in communities that are heterogeneous with reference to the economic background.

Overall it can be concluded that taking into account the heterogeneity of the community, background characteristics, their beliefs, practices, and behaviours are important while implementing large-scale cohort studies. Details on attrition from previous cohort studies in the area are to be considered during the process of implementation. The findings from VTS were pivotal in understanding the community more better and deeper, and some of the observations made were incorporated in the National Surveillance System for Enteric Fever in India (NSSEFI) established in the same area subsequently, the cohort of which is currently under active surveillance [29]. One such important observation incorporated was that, a female FRA was stationed in areas with orthodox families, to do the surveillance. Also, whenever a blood request was made at the clinic by the study physician, the study physician was requested to first explain in detail the reason as to why this particular blood test called *“blood culture”* was being done, the reason for the protocol specified volume of blood and as to why this test takes so long for the results to be reported back.

Hence, strategies in terms of more flexibility in surveillance methods, tailored in the best possible way that does not affect the participants’ nor the families’ sentiments and beliefs might be impactful on cohort retention, although this can prove to be technically challenging with issues of feasibility [30]. Also, innovative yet simple information, education, and communication strategies and tools, keeping the community informed better on research studies can not only go a long way benefitting research but will help the communities appreciating research and its impact on them better. Hence, using tailored strategies specific to the community incorporating the community attitudes and sentiments can be pivotal in strengthening large scale community-based cohort studies. The main limitation of the study was that many of the mothers who withdrew consent form the VTS study did not attend the FGDs following invitation. It is perhaps from these mothers, probably from orthodox backgrounds with whom we could have discussed to understand better on the reasons for withdrawing consent from the study.

## Conclusion

The findings from the drop-out population of Vellore Typhoid Surveillance (VTS) study have highlighted that the heterogeneity of a population and their background, with reference to certain orthodox communities, their socioeconomic status, and type of family being important factors that can affect attrition in a cohort. Also, behavioural aspects, attitudes, and perceptions that exist within the community are important to be considered while implementing large-scale cohort studies, and findings related to reasons for attrition from previous cohort studies are valuable to learn from.

## Data Availability

The VTS study datasets used and analysis described in the current study are available from the corresponding author on reasonable request.

## Abbreviations

CAP: Chinnallapuram
CHAD: Community Health and Development
CMC: Christian Medical College, Vellore
ECR: Eligible Child Register
FGD: Focus Group Discussion
FRA: Field Research Assistant
FRS: Field Research Supervisor
HDSS: Health Demographic and Surveillance System
ICF: Informed Consent Form
LCECU: Low-Cost Effective Care Unit
NSSEFI: National Surveillance System for Enteric Fever in India
RNP: Ramnaickanpalayam
SES: Socio-economic status
VSPM: Vasanthapuram
VTS: Vellore Typhoid Surveillance
